# Exploring the Phospholipid Transport Mechanism of ATP8A1-CDC50

**DOI:** 10.3390/biomedicines11020546

**Published:** 2023-02-13

**Authors:** Honghui Zhang, Yue Zhang, Peiyi Xu, Chen Bai

**Affiliations:** 1 Warshel Institute for Computational Biology, School of Life and Health Sciences, School of Medicine, The Chinese University of Hong Kong, Shenzhen 518172, China; 2 School of Chemistry and Materials Science, University of Science and Technology of China, Hefei 230026, China; 3 Chenzhu (MoMeD) Biotechnology Co., Ltd., Hangzhou 310005, China

**Keywords:** P4-ATPase, ATP8A1-CDC50, phospholipid transport, conformational changes, energy landscape

## Abstract

P4-ATPase translocates lipids from the exoplasmic to the cytosolic plasma membrane leaflet to maintain lipid asymmetry distribution in eukaryotic cells. P4-ATPase is associated with severe neurodegenerative and metabolic diseases such as neurological and motor disorders. Thus, it is important to understand its transport mechanism. However, even with progress in X-ray diffraction and cryo-electron microscopy techniques, it is difficult to obtain the dynamic information of the phospholipid transport process in detail. There are still some problems required to be resolved: (1) when does the lipid transport happen? (2) How do the key residues on the transmembrane helices contribute to the free energy of important states? In this work, we explore the phospholipid transport mechanism using a coarse-grained model and binding free energy calculations. We obtained the free energy landscape by coupling the protein conformational changes and the phospholipid transport event, taking ATP8A1-CDC50 (the typical subtype of P4-ATPase) as the research object. According to the results, we found that the phospholipid would bind to the ATP8A1-CDC50 at the early stage when ATP8A1-CDC50 changes from E2P to E2Pi-PL state. We also found that the electrostatic effects play crucial roles in the phospholipid transport process. The information obtained from this work could help us in designing novel drugs for P-type flippase disorders.

## 1. Introduction

**Figure 1:**
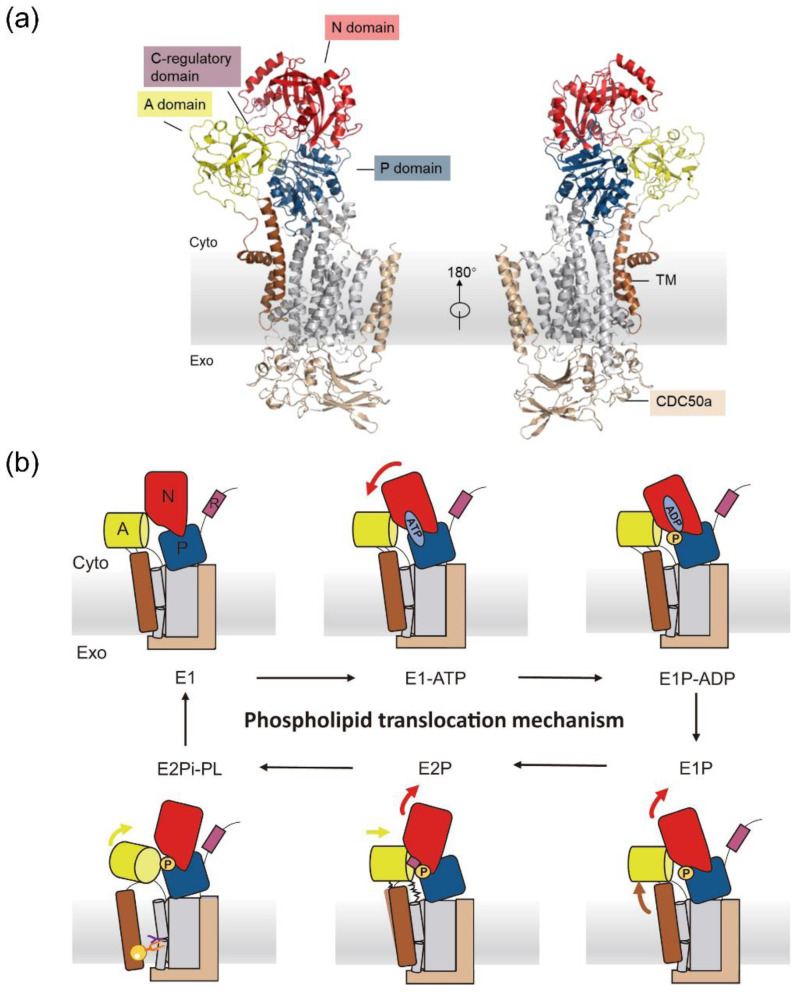
(**a**) Overall structure of P4-ATPase. The A, P, N and C-regulatory domains are colored yellow, blue, red and purple, respectively. TM1, TM2 and TM3–TM10 are dark brown and gray, respectively. The chaperone part CDC50 is in brown. (**b**) Schematic description of the proposed phospholipid transport mechanism for P4-ATPase.

The asymmetric distribution of lipids between the cytoplasmic and exoplasmic leaflets of the biological membrane is a distinctive feature of eukaryotic life [1,2,3]. Phosphatidylserine (PS), phosphatidylethanolamine (PE) and phosphoinositides (PPIns) are mainly located in the inner leaflet, while phosphatidycholine (PC) and sphingomyelin (SM) are concentrated in the extracellular leaflet [4,5]. The asymmetric distribution of lipids plays a critical role in cellular processes such as vesicle formation, blood coagulation, cell signaling and cholesterol homeostasis [3,6]. Disruption of the asymmetric distribution may lead to cell death because of the loss of intercellular interaction [1,7]. P4-ATPase helps to maintain the asymmetry across the membrane by flipping specific lipids from the exoplasmic leaflet to the cytoplasmic leaflet [8,9]. P4-ATPase is a protein typically composed of two parts (Figure 1a) [10]. The first part is the main part with three main cytoplasmic domains (actuator (A) domain, nucleotide-binding (N) domain, phosphorylation (P) domain) and ten transmembrane helices (TM1-TM10). The second part is the chaperoning subunit CDC50 with two transmembrane helices (TM1, TM2) and eight β-sandwiches. How the phospholipids transport happens across P4-ATPase is still under debate. One prevailing hypothesis is the hydrophobic gate model, in which the conduit is composed of TM 1, 2, 4 and 6, and a highly conserved hydrophobic residue I364 (in bovine ATP8A2) acts as a control gate for the phospholipid transport [11]. However, this cannot give an explanation of the phospholipid selectivity and the binding mechanism in the P4-ATPase. Recent advances in the structural biology of P4-ATPase with X-ray diffraction and Cryo-EM experiments have shown us the high-resolution structures of important intermediate states during the phospholipid transport cycle [12,13,14,15]. Six structures of human ATP8A1-CDC50 heterodimer were determined based on cryo-electron microscopy by Hiraizumi et al. [15]. These six important intermediates include: the apo form E1 state, the ATP-analog bound E1-ATP state, the phosphate-analog and ADP bound E1P-ADP state, the transient phosphorylated E1P state, the outward-open E2P state and the phospholipid-bound E2Pi-PL state. The phospholipid transport cycle of P4-ATPase is shown based on the Post-Albers scheme as in Figure 1b [16]. In the E1 state, the protein is free of ligands in the active site. When ATP is bound to the N domain, the N and P domains come close to each other, and the A domain is slightly pushed out. After the ATP hydrolysis, the ADP is released from the N domain, generating a high-energy transient E1P state. Subsequently, the A domain moves close to the N domain, and the C-terminal regulatory domain inserts into the P and N domains, maintaining the stability of the E2P state. TM1 and TM2 are more flexible after the rearrangement of the A domain, which opens the channel and provides the space for the phospholipid bound to the P4-ATPase. The transported phospholipid diffuses into the inner leaflet, and the flippase returns to the apo state, ready for the next transport cycle. The transport mechanism is slightly different from that of P2 ATPase because of the relative rigidity of the TM3-TM10 segment in ATP8A1 [17].

The structural information has revealed the hallmark conformations along the phospholipid transport cycle, but it is insufficient to give energetic and kinetic details during the transport progress. In the computational aspect, the major issue is how to capture the free energy profiles for large and complex systems with limited time and computational resources. Multiscale modeling provides a useful means to estimate the free energy profiles without spending large-scale computational resources [18,19,20]. Here we seek to investigate the progress of phospholipid transport using the coarse-grained (CG) model developed by Arieh Warshel’s group [21,22]. This CG model focuses on improving the description of the electrostatic features and has been validated to provide reliable free energy estimation in many biological systems, including F0-ATPase, GPCRs, myosin, etc. [23,24,25]. The free energy profiles of conformational changes were calculated with the above-mentioned CG model. By using PDLD/SLRA/β method, we also obtained the binding free energy profiles between the phospholipid and P4-ATPase during the phospholipid transport process. The details of the phospholipid-binding mechanism and key residues were revealed, which set the foundations for understanding the phospholipid transport process. Our current study suggests that multiscale modeling can provide useful kinetic information for understanding the mechanism and further guiding drug design.

## 2. Method

**Figure 2:**
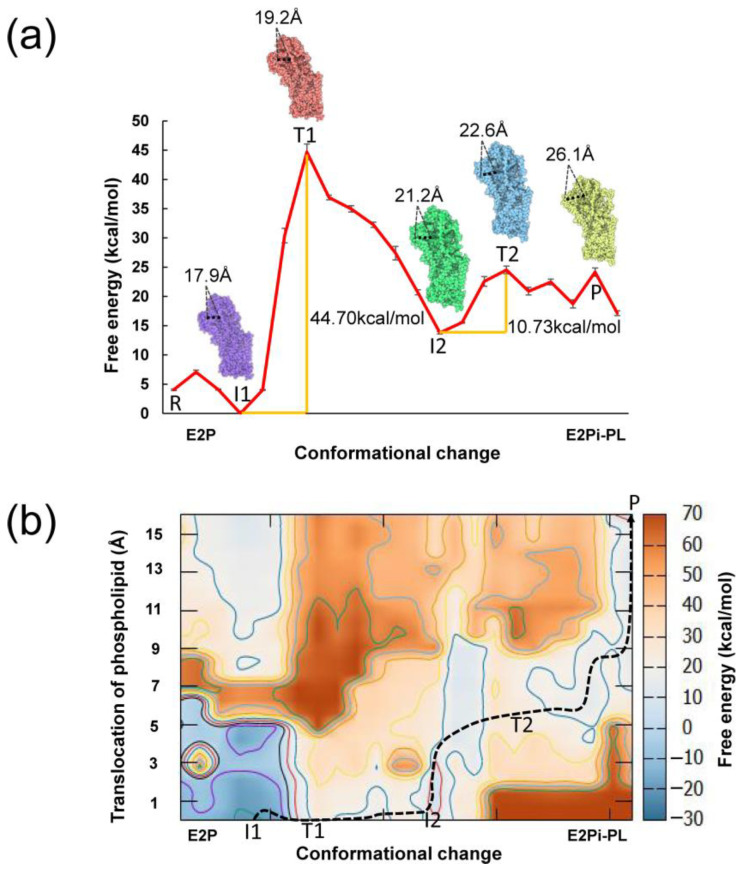
(**a**) The CG free energy profile for the conversion between the E2P and E2PPi-PL states. The representative configurations during the conformational changes were named the reagent state (R), the intermediate structure 1 (I1), the transition structure 1 (T1), the intermediate structure 2 (I2), the transition structure 2 (T2) and the product E2Pi-PL state (P). The energy barriers are shown in orange. The Cα–Cα distances between I121 (TM2) and W376 (TM4) were used to distinguish between the representative conformations (R, I1, T1, I2, T2, P). The distances between atom CA of residue I121 (TM2) and W376 (TM4) for I1, T1, I2, T2 and product structures are shown in the picture, with the value of 17.9 Å, 19.2 Å, 21.2 Å, 22.6 Å and 26.1 Å, respectively (measured with the all-atom structures). This means that the phospholipid transport channel between TM2 and TM4 gradually opens from E2P to the E2Pi-PL state. The free energies were calibrated with the free energy of structure I1. (**b**) Coupled free energy map of the conformational change in P4-ATPase and phospholipid transport. One possible least-energy pathway is indicated by a black dashed line. The barrier along the route is 46.49 kcal/mol.

The initial structures (the outward-open E2P state and the phospholipid-bound E2Pi-PL state) were obtained from the experimental cryo-EM structures resolved by Hiraizumi et al. [15]. The missing segments were repaired by the software Modeller [26]. All the ligands were taken out, and the structures of P4-ATPse were trimmed into the same length. Next, we utilized the target molecular dynamics (TMD) method to construct the conformational changes in P4-ATPase from the E2P to E2Pi-PL state with the all-atom structure in the absence of phospholipid [27]. After obtaining the intermediates from E2P to E2Pi-PL state, we converted these all-atom structures into a CG model and calculated the energy landscape [21,22]. Next, based on the experimental structures resolved by Hiraizumi, we defined the binding pockets of phospholipid [15]. Considering the shortage of the phospholipid-binding structure, the location of the phospholipid was generated from the docking software AutoDock Vina for each intermediate structure. The phospholipid was docked into P4-ATPase by a sophisticated gradient optimization method [28]. Each of the top 15 lipid–protein complexes, based on the scoring function, were extracted to find the best ligand–protein binding mode. The root mean square deviation (RMSD) is 1.306 Å over all the non-hydrogen atoms in the crystal ligand and the docked lipid, which indicates that our docking results are acceptable. In order to calculate the binding free energy, the partial atomic charges of the phospholipid were obtained by the electrostatic potential (esp) calculation with hybrid density functional theory (DFT) at the level b3lyp/6-311g (d,p) [29]. Then, certain characteristics of the phospholipid were incorporated into the Molaris-XG force field, including the names, types and charges of atoms and bond information. PDLD/S-LRA/β method is used to calculate the binding free energy between the phospholipid and P4-ATPase by all-atom structures [30]. By combining these sets of data and the conformational free energy, we could obtain the free energy map that is shown in Figure 2b. The Molaris-XG package was utilized for all relative calculations [22,31].

### 2.1. Coarse-Grained Model

The CG model in this paper simplifies the side chain of each residue with an atom X and a dummy atom D [21]. The atom X usually locates in the geometrical center of the side-chain heavy atoms and shifts towards the ionizable functional atom groups for the ionizable residues, emphasizing the consistent treatment of the electrostatic interaction. The dummy atom D is placed at the boundary of simplified side chain and explicit main chain models, bridging the rotational transformations during the atom’s moving progress. By fitting to the observed absolute stability, the CG model is refined and expresses the total CG free energy as:

(1) $$∆G_{\mathrm{fold}}=∆G_{\mathrm{main}\;}+∆G_{\mathrm{side}}+∆G_{\mathrm{sz}.\mathrm{size}}\;=∆G_{\mathrm{side}}^{\mathrm{elec}}+∆G_{\mathrm{side}}^{\mathrm{polar}}+∆G_{\mathrm{side}}^{\mathrm{hyd}}+c_{1}∆G_{\mathrm{side}}^{\mathrm{vdw}}+c_{2}∆G_{\mathrm{solv}}^{\mathrm{CG}}+c_{3}∆G_{\mathrm{HB}\;}^{\mathrm{CG}}+∆G_{\mathrm{sz}.\mathrm{size}}^{\mathrm{vdw}}$$

where $∆G_{\mathrm{main}}\;\mathrm{and}\;∆G_{\mathrm{side}}$ represent the main-chain and side-chain contribution, respectively. $∆G_{\mathrm{sz}.\mathrm{size}}$ denotes the effect of the whole protein and side-chain flexibility on energy. $∆G_{\mathrm{side}}^{\mathrm{vdw}}$, $∆G_{\mathrm{side}}^{\mathrm{elec}},\;∆G_{\mathrm{side}}^{\mathrm{polar}}\;\mathrm{and}\;∆G_{\mathrm{side}}^{\mathrm{hyd}}$ are the electrostatic, polar, hydrophobic and Van der Waals components of the side chain. $c_{1}$ is a scaling coefficient with a value of 0.10. $∆G_{\mathrm{solv}}^{\mathrm{CG}}\;\mathrm{and}\;∆G_{\mathrm{HB}}^{\mathrm{CG}}$ represent the contributions of the main-chain solvation and the hydrogen bonds. $c_{2}$ and $c_{3}$ are scaling coefficients, with values of 0.25 and 0.15, respectively. The constants $c_{1}$, $c_{2}$ and $c_{3}$ are experience parameters, and the value set in the current implementation has been proven to be effective in researching the relationship between structure and function in large macromolecular complexes [18,32]. $∆G_{\mathrm{sz}.\mathrm{size}}^{\mathrm{vdw}}$ is the Van der Waals component for main-side interactions. The emphasis of the CG model is on the electrostatic term $∆G_{\mathrm{side}}^{\mathrm{elec}}$ which is given by:

(2) $$∆G_{\mathrm{side}}^{\mathrm{elec}}=-2.3\mathrm{RT}\sum_{i}Q_{i}^{\mathrm{MC}}\left({pKa}_{i}^{b}-\;{pKa}_{i}^{w}\right)+∆G_{\mathrm{QQ}}^{\left(f\right)}-\;∆G_{\mathrm{QQ}}^{\left(\mathrm{uf}\right)}+∆G_{Q}^{\mathrm{dev}}$$

In this formula, $Q_{i}^{MC}$ represents the Monte Carlo averaged charge carried by i*th* residue in the given ionization state. ${pKa}_{i}^{b}$ represents the ${pK}_{a}$ in the protein environment for i*th* ionizable residue. ${pKa}_{i}^{w}$ are the ${pK}_{a}$ in water for i*th* ionizable residue [32]. $∆G_{QQ}^{\left(f\right)}$ and $∆G_{QQ}^{\left(uf\right)}$ are the charge-charge interaction potential in the folded and unfolded protein. $∆G_{Q}^{dev}$ describes the penalty of changing the protonation state of an ionizable residue upon unfolding.

An important part of the CG model is the estimation of ${pKa}_{i}^{b}$ by the Monte Carlo Proton Transfer (MCPT) method. The ionization states of the residues under the specific PH and temperature T are determined by the Metropolis Monte Carlo (MC) approach [21,33]. A pair of ionizable residues or one ionizable residue and the bulk solvent would be chosen to conduct an attempt for proton transfer (PT) in each MC step. This iteration does not stop until the electrostatic free energy of the folded protein converges; then, the ionization states of the protein residues are obtained to evaluate the CG free energy. In each MC move, the electrostatic force of a folded protein for the m-th charge configuration $Q_{i}^{\left(m\right)}$ is calculated as:

(3) $$∆G_{\mathrm{elec}}^{\left(m\right)}=-2.3\mathrm{RT}\sum_{i}Q_{i}^{\left(m\right)}\left({pKa}_{i}^{b}-\mathrm{pH}\right)+∆G_{\mathrm{QQ}}^{\left(m\right)}$$

In which $∆G_{\mathrm{QQ}}^{\left(m\right)}$ represents the charge interaction free energies for the m*th* charge configuration. The charge configuration is accepted when a lower value of electrostatic free energy is achieved or the Met–chargeropolis criteria are met. The minimized $∆G_{\mathrm{elec}}$ is subsequently utilized to estimate the electrostatic contribution to the folding free energy by Equation (2). The ${pKa}_{i}^{b}$ values of the ionizable residues are given by:

(4) $${pKa}_{i}^{b}={pKa}_{i}^{w}-\;\frac{\mathrm{sgn}(Q_{i}^{\mathrm{ion}})}{2.3\mathrm{RT}}∆G_{\mathrm{self},i}$$

In this model, $\mathrm{sgn}\left(Q_{i}^{\mathrm{ion}}\right)$ represents the sign function of the charge for the i*th* residue, with a value of +1 for Arginine, Lysine and Histidine and −1 for Aspartic Acid and Glutamic acid. $∆G_{\mathrm{self},i}$ means the changes for self-energy, an ionizable residue moving from water to the protein. This term is associated with charging every ionizable functional group in the given environment. The term $∆G_{\mathrm{self},i}$ is computed by:

(5) $$∆G_{\mathrm{self},i}=\sum_{i}[U_{p}^{\mathrm{self}}\left(N_{p}^{i}\right)+U_{\mathrm{np}}^{\mathrm{self}}\left(N_{\mathrm{np}}^{i}\right)+U_{\mathrm{mem}}^{\mathrm{self}}\;(N_{\mathrm{mem}}^{i})]$$

Here, U means effective potential. When dealing with membrane proteins, we represent the membrane using a grid made up of uniform atoms. The membrane grid particles in the vicinity of protein atoms and within the protein are not being constructed. The self-energy contribution of non-polar residues, polar residues and membrane grid points are represented by $U_{p}^{\mathrm{self}},\;{\;U}_{\mathrm{np}}^{\mathrm{self}}\;\mathrm{and}\;U_{\mathrm{mem}}^{\mathrm{self}}$, respectively. The number of regarding residues (non-polar, polar and membrane atoms around the i*th* residue) is given by $N_{p}^{i},\;N_{\mathrm{np}}^{i}\;\mathrm{and}\;N_{\mathrm{mem}}^{i}$, respectively. According to the previous attempts by Rychkova et al., the distance between the solvent and membrane grids is important in the self-energy estimation [34]. An alternative that was once considered was utilizing the Langevin dipoles grid; however, this approach would have resulted in an overlap of the self-energy penalty in the middle of the membrane [35]. Thus the self-energy contribution of membrane grid points $U_{\mathrm{mem}}^{\mathrm{self}}$ is given by:

(6) $$U_{\mathrm{mem}}^{\mathrm{self}}=\left\{\begin{array}{c} U_{\mathrm{mem}}^{\mathrm{self},\mathrm{ion},0}exp\left[-({(R_{\min\;}-\;18)/12}^{2}\right]\;\;\;\;\;\;\;R_{\min}\;\leq \;18\\ U_{\mathrm{mem}}^{\mathrm{self},\mathrm{ion},0}\;\;\;\;\;\;\;\;\;\;\;\;\;\;\;\;\;\;\;\;\;\;\;\;\;\;\;\;\;\;\;\;\;\;\;\;\;\;\;\;\;\;\;\;\;\;\;\;\;\;\;\;\;\;\;R_{\min\;}\leq \;18 \end{array}\right.\;U_{\mathrm{mem}}^{\mathrm{self},\mathrm{ion},0}=\left\{\begin{array}{c} B_{\mathrm{mem}}^{\mathrm{self},\mathrm{ion}}exp\left[-0.2(N_{\mathrm{mem}}-\;28)\right]\;\;\;\;\;\;\;{0\;<\;N}_{\mathrm{mem}\;}\leq 28\\ B_{\mathrm{mem}}^{\mathrm{self},\mathrm{ion}}\;\;\;\;\;\;\;\;\;\;\;\;\;\;\;\;\;\;\;\;\;\;\;\;\;\;\;\;\;\;\;\;\;\;\;\;\;\;\;\;\;\;\;\;\;\;\;\;\;\;\;\;\;\;\;\;\;\;N_{\mathrm{mem}}\;>\;28 \end{array}\right.$$

Here, $R_{\min}$ means the length from the current residue to the closest solvent molecule. $B_{\mathrm{mem}}^{\mathrm{self},\mathrm{ion}}$ has a value of 15, taken from the experimental data and computational simulations, with the unit as kcal/mol [30]. $N_{\mathrm{mem}}$ denotes the number of surrounding membrane points. Therefore, when the protein approaches the grid points, their interaction continuously diminishes. This continuous membrane model lessens the requirement for creating a fresh grid when the protein moves or alters its structure. At the same time, the partial double counting of the self-energy penalty in the middle of the membrane is avoided. This membrane-treating method has been proven to be suitable for many transmembrane systems [23,32,36]. Through these calculations, we can obtain the conformational free energy. It is worth noting that free energy perturbation (FEP) is not involved in our current free energy calculations because our coarse-grained model takes into account the apparent free energy and the evaluation of the effective potential [30].

### 2.2. PDLD/S-LRA/β

PDLD/S-LRA/β method is a combination of linear response approximation (LRA) and the semi-microscopic version of the protein–dipole Langevin–dipole (PDLD/S) with a scaled non-electrostatic Van der Waals term [37]. PDLD/S-LRA/β provides fast qualitative estimates of binding energy between ligands and proteins. The effective PDLD/S potential for the lipid-bound/unbound to P4-ATPase is calculated by:

(7) $${U_{\mathrm{elec},l}^{P}=\left\{\left({∆G}_{\mathrm{sol}}^{l+p}-\;{∆G}_{\mathrm{sol}}^{l^{’}+P}\right)\left(\frac{1}{\varepsilon_{P}}-\;\frac{1}{\varepsilon_{W}}\right)+{∆G}_{\mathrm{sol}}^{l}\left(1\;-\;\frac{1}{\varepsilon_{P}}\right)+\frac{U_{q\mu }^{l}}{\varepsilon_{P}}+\frac{U_{\mathrm{intra}}^{l}}{\varepsilon_{P}}\right\}}_{B}\;{U_{\mathrm{ele},l}^{W}=\left\{{∆G}_{\mathrm{sol}}^{l}\left(\frac{1}{\varepsilon_{P}}-\;\frac{1}{\varepsilon_{w}}\right)+{∆G}_{\mathrm{sol}}^{l}\left(1\;-\;\frac{1}{\varepsilon_{P}}\right)+\frac{U_{\mathrm{intra}}^{l}}{\varepsilon_{P}}\right\}}_{\mathrm{UB}}$$

Here, p and l represent the protein and the ligand, respectively. $l^{’}$ is the uncharged state of ligand. $∆G_{sol}^{i}$ means the solvation energy of protein or ligand atoms in water. $\varepsilon_{P}$ and $\varepsilon_{W}$ are the dielectric constant of protein and water, respectively. $U_{q\mu }^{l}$ is the electrostatic contribution between the charges carried by ligand (q) and the protein dipoles (μ) under vacuum. $U_{intra}^{l}$ is the intrinsic electrostatic energy of ligand. Subsequently, the protein reorganization is captured by LRA [38]. Thus, the electrostatic contribution of the binding energy $∆G_{bind}^{elec}$ is given by:

(8) $$∆G_{\mathrm{bind}}^{\mathrm{elec}}=\frac{1}{2}\left[{\langle U_{\mathrm{elec},l}^{P}\rangle }_{l}+{\langle U_{\mathrm{elec},l}^{P}\rangle }_{{l\;}^{’}}-\;{\langle U_{\mathrm{elec},l}^{W}\rangle }_{l\;}-\;{\langle U_{\mathrm{elec},l}^{W}\rangle }_{l^{’}}\right]$$

In which the sign < > means the average of specific effective potential denoted by U. ${\langle U_{\mathrm{elec},l}^{P}\rangle }_{l^{’}}$ is the average effective potential over a protein configuration generated under the protein force field. The binding free energy is calculated by:

(9) $${∆G}_{\mathrm{bind}}^{\mathrm{PDLD}/s-\mathrm{LRA}/\beta }={∆G}_{\mathrm{bind}}^{\mathrm{elec}}+\beta \left[{\langle U_{\mathrm{vdw},l}^{P}\rangle }_{l}-\;{\langle U_{\mathrm{vdw},l}^{W}\rangle }_{l}\right]$$

The non-electrostatic Van der Waals part is scaled with the β equaling 0.25, which is obtained by fitting with the experimental observations [37]. With this calculation step, the binding free energy between the phospholipid and P4-ATPase was obtained.

### 2.3. Targeted Molecular Simulations (TMD)

The intermediate structures between E2P and E2Pi-PL were generated by targeted molecular simulations (TMD). TMD is widely used to simulate large systems over long time scales, providing information about the long-term behavior of the system [27]. TMD simulations can produce conformational transition paths within a very short simulation time, which may not be sufficient to capture all relevant biological processes [39,40]. However, TMD simulations can provide a comprehensive and concrete representation of the conformational changes and transition processes in proteins. In each step of TMD simulations, the RMS distance between the initial coordinates and the final structure is calculated. The driving force on each atom is obtained regarding the gradient of the potential:

(10) $$U_{\mathrm{TMD}}=\frac{1}{2}\frac{k}{N}{\left[\mathrm{RMS}\left(t\right)-\;\rho \left(t\right)\right]}^{2}$$

Here N is the atom number of the whole system. k refers to the spring constant. $RMS\left(t\right)$ describes the best-fit root mean square deviation between the structure and the target configuration at the moment t. ρ(t) is a reference RMSD value at time t. The rms distance for the whole system from its current position to the target is shrinking monotonously during the TMD simulation, driving the moving structure toward the target configuration [27]. For each domain, forces are computed separately from other domains. In this work, k was set as 100.0 kcal/mol/Å^2^ to avoid the system collapse caused by a too-large value setting and also to avoid the insufficient deformation caused by too little value setting [41]. Twenty-one structures were picked at equal intervals to reproduce the free energy profiles in Figure 2.

## 3. Results

### 3.1. Coarse-Grained Free Energy Profile for Conformational Changes

In this work, we focused on the transition from the phosphoenzyme state E2P to the dephosphorylation state E2Pi-PL. The E2P state was ready to bind to the phospholipid from the outer leaflet, while the E2Pi-PL state was bound to the phospholipid. The initial structures of the P4-ATPase were obtained from the protein data bank (PDB id: 6K7L for E2P, 6K7M for E2Pi-PL) [42]. The missing loops and helixes were added by the program Modeller [26]. The crystal water, ligand and inhibitors from cryo-EM structures were removed. The targeted molecular dynamics (TMD) method was used to generate a series of intermediate structures between the E2P state and the E2Pi-PL state. The entire CG free energy profile from the phospholipid-free state to the phospholipid-bound state is presented in Figure 2a. The representative structures during the conformational changes were named the reagent state (R), the intermediate structure 1 (I1), the transition structure 1 (T1), the intermediate structure II (I2), the transition structure 2 (T2) and the product E2Pi-PL state (P). The free energy of the P state is higher than the R state. During the transport of phospholipids, P4-ATPase utilizes the energy generated from the hydrolysis of ATP to move phospholipids from the extracellular side to the cytoplasmic leaflet [43,44,45]. That means it is not an isolated system. This energy source from ATP hydrolysis enables the transfer of phospholipids across the lipid bilayer and may explain why the final state has high energy. The experimentally comparable parameters are still limited even for the sodium-potassium ATPases and calcium ATPases within the same family [44,46,47,48]. From the structural perspective, the channel between TM2 and TM4 opens to adapt to the binding of phospholipids during the conformational change from E2P to E2Pi-PL state [15,49]. Newly formed space within the P4-ATPase between the transmembrane helices may partly cause the breakage of the protein interaction network, thus decreasing the stability of the P4-ATPase. These intrinsic changes may cause a higher free energy value of the P state compared with the R state. The energy barriers of the conformational changes were 44.70 and 10.73 kcal/mol (Figure 2a). The highest barrier occurs from I1 to T1, and it is 44.70 kcal/mol. For P4-ATPase, due to the complexity and the uncertainty of the exact nature of the phospholipid transport process, it is challenging to generate the detailed energy landscape of the phospholipid transport mechanism [50,51]. The energy barrier for conformational change in other ATPase members, such as F1-ATPase and Adenosine triphosphate (ATP)-binding cassette (ABC) transporters, varies significantly, ranging from 10 to 32.1 kcal/mol [52,53,54]. The relatively high barrier in our results may be a result of the large molecular conformation and specialized function of phospholipid transporters [55]. The second energy barrier is 10.73 kcal/mol during the I2–T2 transition, which is caused by the hydrophilic pathway for the hydrophilic phospholipid head group translation during the phospholipid transport [56]. The P4 ATPase protein directly contacts the polar head group of the phospholipid, so a wide hydrophilic pathway is required [57,58].

**Figure 3:**
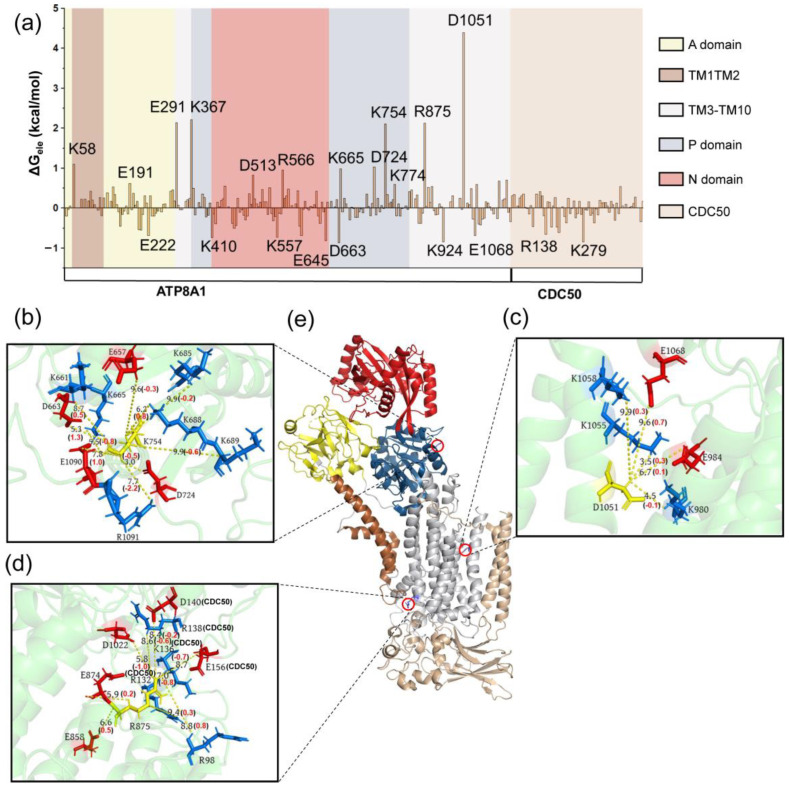
(**a**) The electrostatic contribution of residues for structures I1 and T1. The values in parentheses indicate the distance changes in specific residues in the T1 and I1 structures. The interaction map of K754 (**b**), D1051 (**c**), R875 (**d**) and their corresponding position in the P4-ATPase (**e**). The positively charged residues are colored blue, and the negatively charged residues are shown in red. The values in parentheses are the distance difference between the T1 and I1 structures.

**Figure 4:**
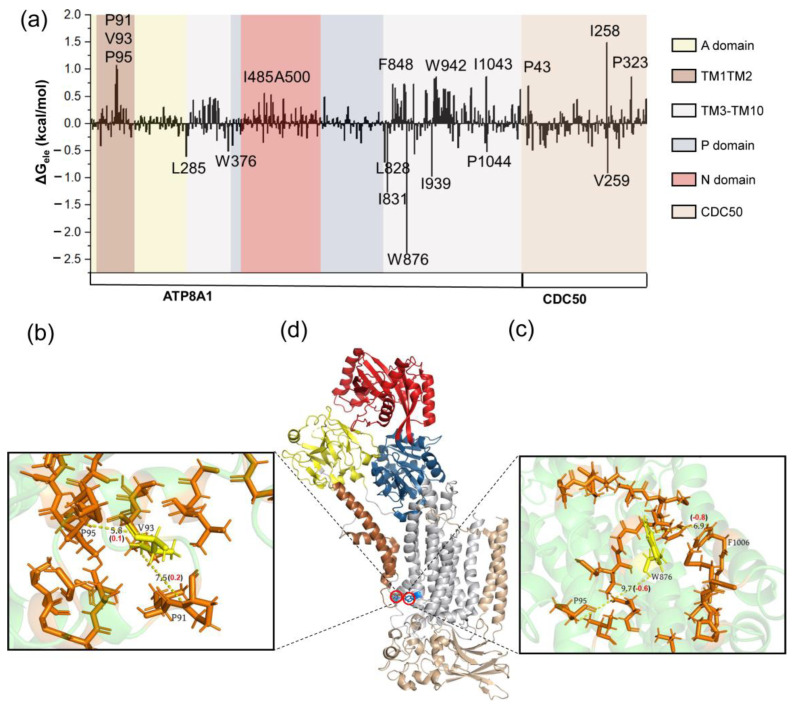
(**a**) The hydrophobic contribution of residues for structures I1 and T1. The interaction map of V94 (**b**), W876 (**c**) and their corresponding position in the P4-ATPase (**d**). The hydrophobic residues are colored orange.

**Figure 5:**
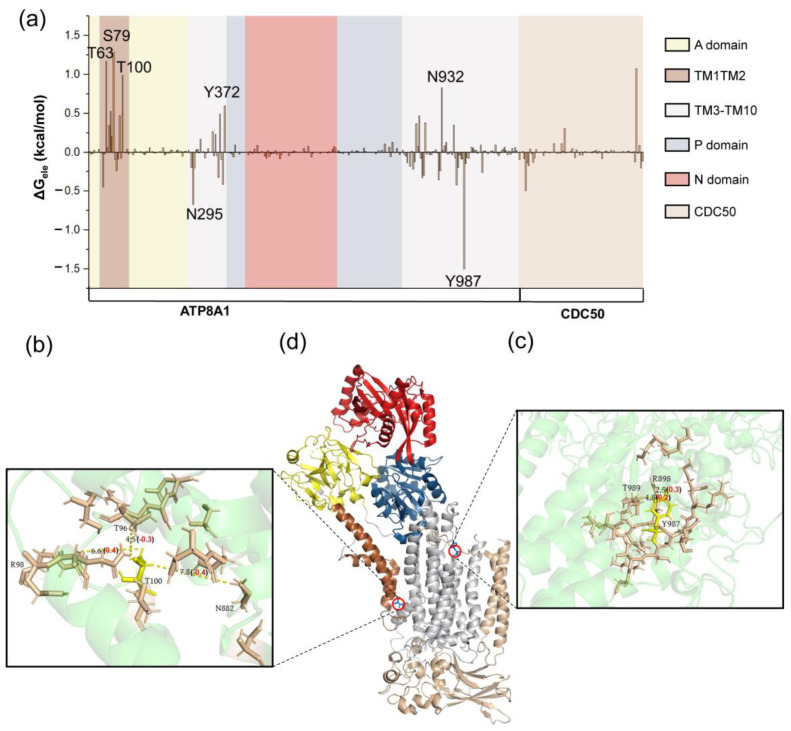
(**a**) The polar contribution of residues for structures I1 and T1. The interaction map of T100 (**b**), Y987 (**c**) and their corresponding position in the P4-ATPase (**d**). The polar residues are colored wheat.

The energy decompositions for these representative states are listed in Table S1. We found that the electrostatic, hydrophobic and polar energy terms play an important role for the first energy barrier, while the electrostatic interaction mainly contributes to the second barrier. The energy contribution and structural information of the key residues of P4-ATPase are presented in Figure 3, Figure 4 and Figure 5 (from I1 to T1) and 6 (from I2 to T2). From I1 to T1 structure, the key residues D1051, R875 and K754 (these residues are not very sequence-conserved in other P4-ATPase; Figure S1) are mainly located in the TM3-TM10 and P domain (Figure 3a). The current results indicate that the sequence conservation of important residues is low. In the future, we will perform site-directed mutagenesis experiments to validate the computational predictions. This will allow us to establish a direct link between the sequence variations and the function of the P4-ATPases. At the same time, we will further analyze the influences on different translocation stages. For the negatively charged residue D1051, the surrounding residues are mainly moving away from it during the conformational changes from I1 to T1, with the distance change 0.7 (E1068), 0.3 (K1058), 0.3 (K1055) and 0.1 (E984) Å from I1 to T1 structure (Figure 3d). These findings indicate that although these residues are not within the binding site, their minor movements of the backbone induce changes in the whole protein interaction network. In our results, the region CDC50 plays an indispensable role, especially for R875 (Figure 3e). The positively charged residues K136, R138, D140 and E156 located in the CDC50 region move closer to R875. The electrostatic repulsion induced by these positively charged residues may lead to a higher energy barrier from I1 to T1 [59].

**Figure 6:**
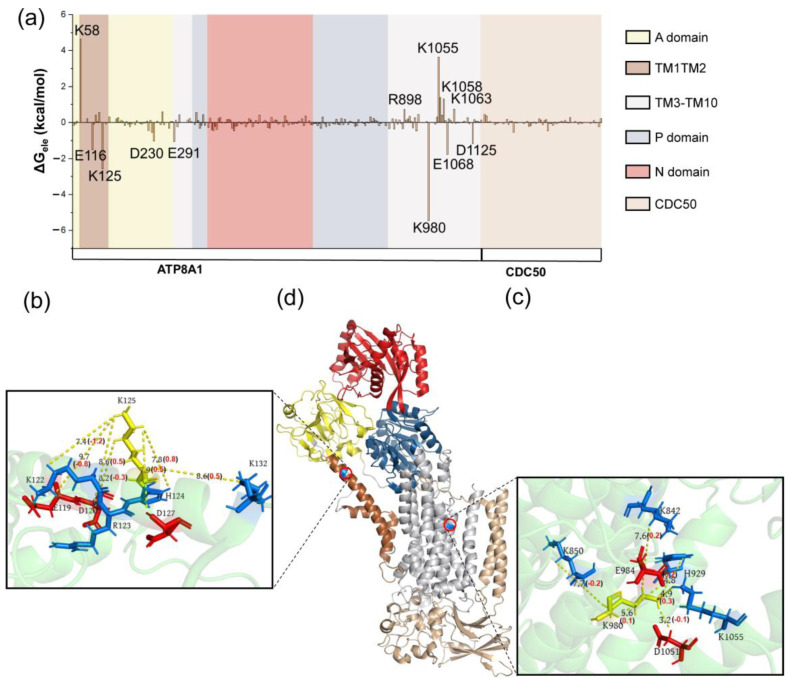
(**a**) The electrostatic contribution of residues for structures I2 and T2. The interaction map of K125 (**b**), K980 (**c**) and their corresponding position in the P4-ATPase (**d**). The positively charged residues are colored blue, and the negatively charged residues are shown in red.

The residues in the main part of the protein collectively contribute to the hydrophobic energy item, as shown in Figure 4a. The hydrophobic residues (V93, W876) are located near the phospholipid binding site (Figure 4b–d) and play an important role in the conformational changes. These residues may help the arrangement of the acyl chains of the phospholipid tail during the phospholipid transport [56]. Different from the results of hydrophobic energy decomposition, the residues located in the transmembrane part (TM1-TM10) play a dominant role in the polar interactions (Figure 5a). For T100, polar residues N882 and T96 move closer to it, while R98 moves away from it. These movements positively contribute to the energy barrier from I1 to T1 (Figure 5b). Nakanishi and his coworkers found that the mutation in T90 in ATP11C (another human P4-ATPase subtype), corresponding to T100 in our work, substantially affects ATPase activity in the mutational experiments [14]. T90 in ATP11C may take part in the hydrogen bond formation with the polar head group of the phospholipid. Those observations are consistent with our finding that conservative polar residue T100 in our work is indispensable for phospholipid transport. In terms of energy and structure, the movements of Y987 and the surrounding residues R898, T989 are in favor of the conformational changes from I1 to T1 (Figure 5d). As for the electrostatic contribution from I2 to T2 state, the critical residues are mainly located in TM1-TM10, with large conformational changes (Figure 6). According to the findings of Timcenko et al., the lateral rearrangement of TM1-TM2 is obvious, while TM3-TM10 is a structurally stable region throughout the phospholipid transport cycle [14,15,57,60]. Our findings suggest that even small movements of the residues in the structural stable region can lead to the changes in the interaction network, which further affects the phospholipid transport.

### 3.2. Conformational Changes Coupled with the Phospholipid Transport

Despite immense structural advances in recent years, there are still puzzles about how the phospholipid binds to the outer leaflet of P4-ATPase and translates towards the cytoplasmic leaflet [61,62,63]. Moreover, how the translation is coupled with the conformational change in the protein is unclear. In this work, we obtained the free energy surface that couples phospholipid transport events and conformational change in the protein.

The coupling energy surface is depicted in Figure 2b. The energy surface illustrates how the phospholipid binds and transports along with the conformational changes. There is an energy favorable point I1 at the beginning of conformational changes from E2P to E2Pi-PL state (Figure 2b and Figure S2). The energy barriers of the coupling energy map were 46.49 and 16.83 kcal/mol (Figure S2). At point I1, P4-ATPase binds with the phospholipid. It is unlikely that the phospholipid is transported directly at the beginning without conformational changes due to the relatively high energy barrier (Figure S2). Once reaching the T2 structure, there is no more dramatic change in the energy curve. The phospholipid transport can occur only when the conformational change reaches a certain point when the channel is half-open (Figure 2b). Without the conformational change, the phospholipid cannot be transported.

**Figure 7:**
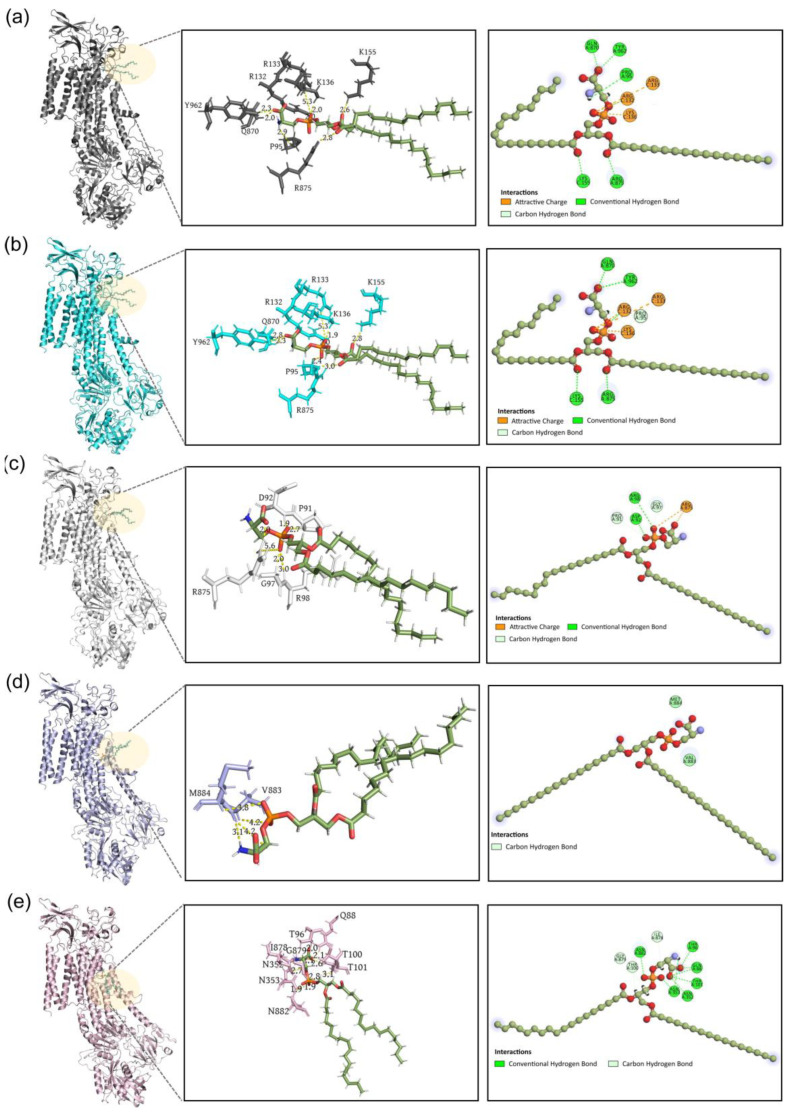
The lipid–protein interactions and the corresponding interactions on the 2D diagram for (**a**) I1 structure (colored dark grey), (**b**) T1 structure (colored cyan), (**c**) I2 structure (colored light grey), (**d**) T2 structure (colored purple) and (**e**) P state (colored pink). For each state, the left panel represents the 3D structure of the complex; the middle panel represents the lipid–protein interactions; the right panel represents the interactions on the 2D diagram.

To investigate the interaction details, we analyzed the lipid–protein interactions of I1, T1, I2, T2 and P states by Discovery Studio [64] (Figure 7). The P4-ATPase binds tightly to the phospholipid for I1 and T1 structures (Figure 7a,b). The positively charged residues R132, R133, K136 and K155 in CDC50 play a role in phospholipid binding by the electrostatic attraction and hydrogen bond interactions. P95, Q870, R875 and Y962 form hydrogen bond networks with the polar head of the phospholipid. From I1 to T1, several residues move away from the phospholipid, with the distances 0.2 Å for R875 and Y962 and 0.5 and 0.3 Å for K155. For P95, even though the side chain of this hydrophobic residue moves closer to the oxygen atom of the phospholipid, the hydrogen bond becomes weaker compared with the hydrogen bond interaction in the I1 structure (Figure 7a,b). Even with subtle movements from I1 to T1 structure, the lipid–protein binding mode is highly similar. We discovered the important residues R132, R133, K136 and K155 in CDC50, which form hydrogen bonds or electrostatic attractions with the polar head of phospholipid (Figure 7a,b). These residues have also been reported in Cryo-EM and X-ray diffraction experiments [15,60].

After reaching the I2 structure, R875 rotates and forms the electrostatic attraction interaction with the phospholipid (Figure 7c). There are relatively strong hydrogen bonds between the phospholipid and P4-ATPase. Once reaching the T2 structure, the interactions between the phospholipid and its surrounding environment become weak (Figure 7d). V883 and M884 form hydrogen bonds with the phospholipid. For the P state, there are several relatively strong hydrogen bonds without significant electrostatic attraction (Figure 7e). Protein–lipid interaction plays an important role in the energetics of P4-ATPase, as it can affect the protein’s conformational changes and electrostatic properties. These interactions can affect the protein’s ability to transport phospholipids, which has important implications for the cell’s physiology. On the basis of the results, we found that electrostatic interactions play an important role in phospholipid transport. The electrostatic interactions of P4-ATPase can affect the orientation and stability of P4-ATPase, and thus affect the activity of P4-ATPase. Our results are consistent with the point that electrostatic effects have a decisive influence on the function of biomolecules [65,66]. It has been demonstrated that electrostatic interaction plays a critical role in membrane transport [67], enzyme catalysis [68] and ligand binding [69]. Electrostatic interactions provide a positively charged environment to the acidic headgroup of the phospholipid.

## 4. Discussions

Electron microscopy and X-ray diffraction can provide structural information about P4-ATPase, but the kinetic details of the phospholipid transport and conformational changes are difficult to obtain. Computer simulations can effectively deal with these problems. We constructed the coarse-grained model and obtained the free-energy landscape of the conformational changes in ATP8A1-CDC50. The two conformational barriers were estimated as 44.70 and 10.73 kcal/mol, respectively. The energy decomposition analysis results indicate that the transmembrane domain, TM1-TM10, is responsible for phospholipid transport by contributions from electrostatic, hydrophobic and polar interactions. On the other hand, it was reported that residues contributing to phospholipid transport would be located near the phospholipid head group [70]. The TM3-TM10 region, which is regarded as the structural stable part during the phospholipid transport [15], contributes a lot to the electrostatic and hydrophobic interactions. The movements of the bulk residues, such as K980 and D1051, may result in substantial influence on the P4-ATPase interaction network.

Next, as presented in Figure 2b, the conformational free energy profiles are coupled with the phospholipid binding free energy, and the three-dimensional energy map is obtained. The phospholipid is unable to transport at the early stage until major conformational change occurs. It has been reported that CDC50 plays a crucial role in protein folding and the formation of activating a state of P4-ATPase [71]. P4-ATPase could not transport the phospholipids without the CDC50 region expression, according to heterologous expression studies [72]. Based on binding modes analysis, we found that the region CDC50 participates in the transport cycle of P4-ATPase with electrostatic effects and promotes the binding of the phospholipid. R132, R133, K136 and K155 located in CDC50 region, together with P95, Q870, R875 and Y962 in ATP8A1 region, form the cavity to accommodate the polar head group of phospholipids. Similar findings have been reported in yeast Drs2p, where the CDC50 was involved in creating a high-affinity phospholipid-binding site [73,74]. The indispensable role of electrostatic effects is restated for the phospholipid transport of P4-ATPase, just as it has been presented in many biological systems such as GPCR and GSDMD [24,67].

**Figure 8:**
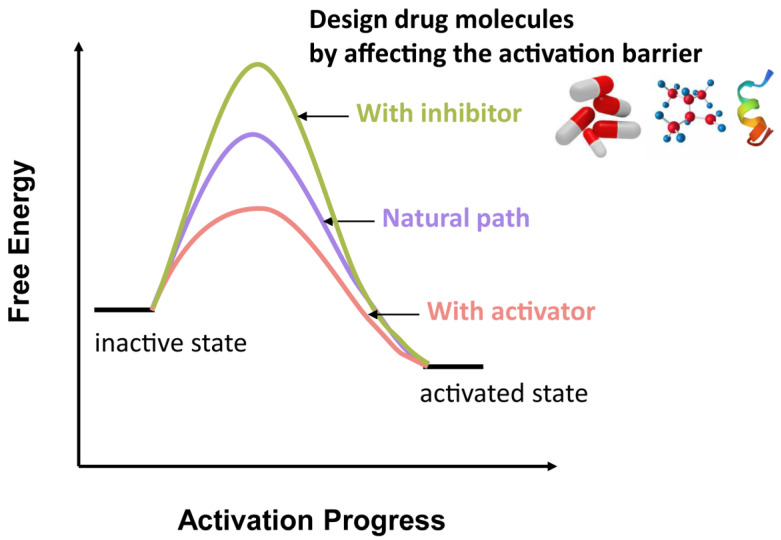
The drug design strategy by affecting the barrier of the protein activation process.

Our study helps in understanding how multiple events are correlated to each other and explains the mechanism details at the atomic level. These findings could complement the static structures obtained from X-ray or cryo-EM experiments. The elucidation of the transition and intermediate states of the phospholipid transport cycle provides important kinetic information for novel drug design on P4-ATPase. By intervening in the energy barriers of the activation process, we could design the inhibitors or activators to treat diseases caused by the dysfunction of the P4-ATPase (Figure 8). When designing activators, the energy barrier needs to be decreased. When designing inhibitors, the energy barrier needs to be increased. Implementing this strategy requires the determination of an accurate least energy pathway and transition state structure. Our work, however, provides a reasonable approach to obtaining such information to be used in future drug design. Understanding the energy barriers of phospholipid transporter can have important pharmacological applications for the development of drugs that modulate the activity of phospholipid transporters. It has therapeutic benefits for disorders such as Parkinson’s disease, myocardial infarction and non-small cell lung cancer et al. [75,76].

## 5. Conclusions

In this work, the consistently developed coarse-grained model was used to investigate the phospholipid transport of the large-size transmembrane protein P4-ATPase from the E2P state to the E2Pi-PL state. We obtained the free energy surface of conformational changes coupled with the phospholipid transport, the least energy pathway and the barrier of the process. The transmembrane helices TM3-TM10 are relatively rigid in other researchers’ work [15]. According to the analysis coupling phospholipid transport and conformational change, we found that residues such as K980 and K1055, located in TM3-TM10, play an important role in the energy contribution. Our findings indicate that the transmembrane domain, TM1-TM10, plays an important role in phospholipid transport. The polar binding site was recognized, which is formed by R132, R133, K136 and K155 in the region CDC50 of P4-ATPase, as well as P95, Q870, R875 and Y962 in the region ATP8A1. The electrostatic effects were found to promote phospholipid binding and transport. Our study helps to unravel the microscopic mechanism and provides valuable kinetic information that could help in future P4-ATPase drug design.

## Data Availability

All data generated and analyzed during this study are included in this article.

## Supplementary Materials

The following support information can be downloaded at https://www.mdpi.com/article/10.3390/biomedicines11020546/s1: Figure S1: Sequence alignment of ATP8A1 and other P4-ATPase. Figure S2: The coupling energy of the system during the conformational changes. Table S1: The energy decomposition of I1, T1, I2, T2, P state (unit: kcal/mol).
