# Supplementary material for: Exploring the Phospholipid Transport Mechanism of ATP8A1-CDC50
Source: Biomedicines. 2023 Feb 13;11(2):546. doi: 10.3390/biomedicines11020546 (PMC9953615; doi:10.3390/biomedicines11020546)
Supplement: Supplementary file 1 [file biomedicines-11-00546-s001.zip › biomedicines-2157594-supplementary.pdf]

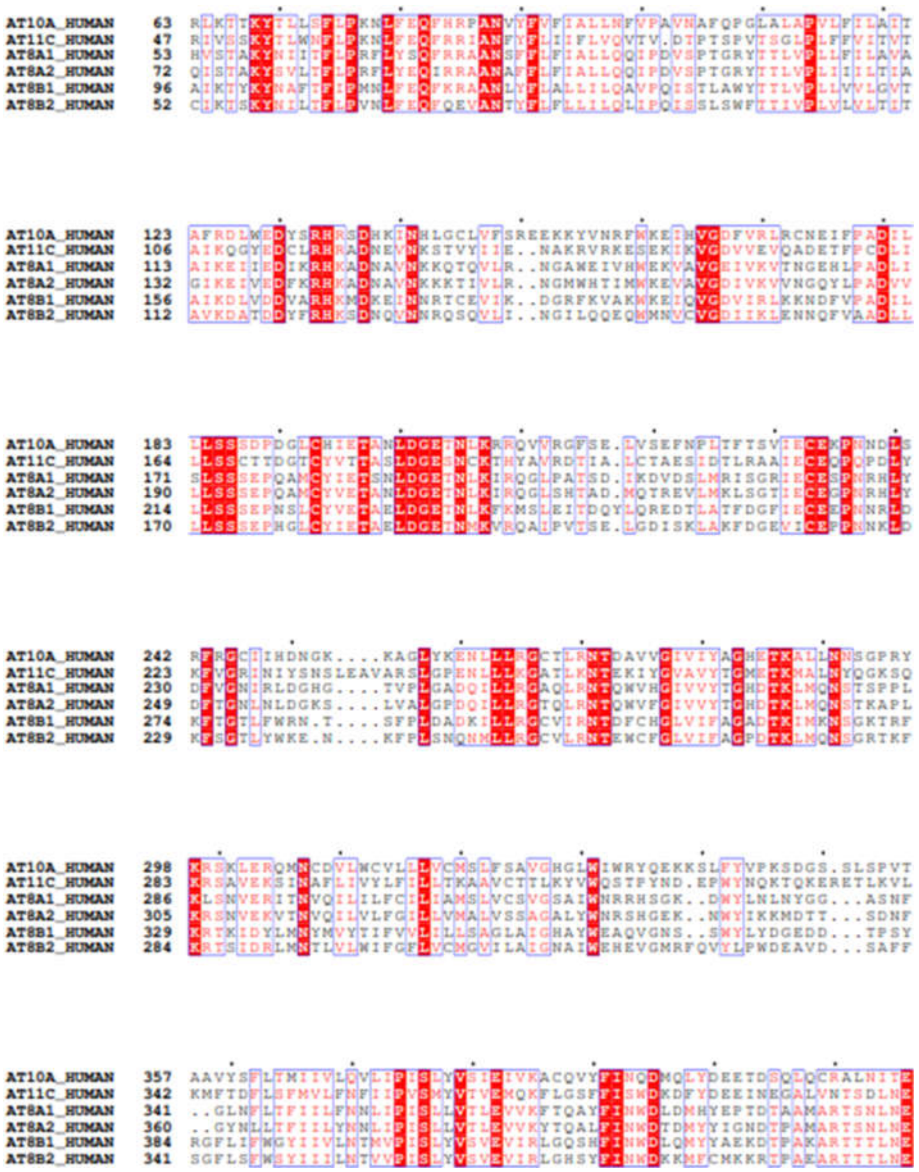

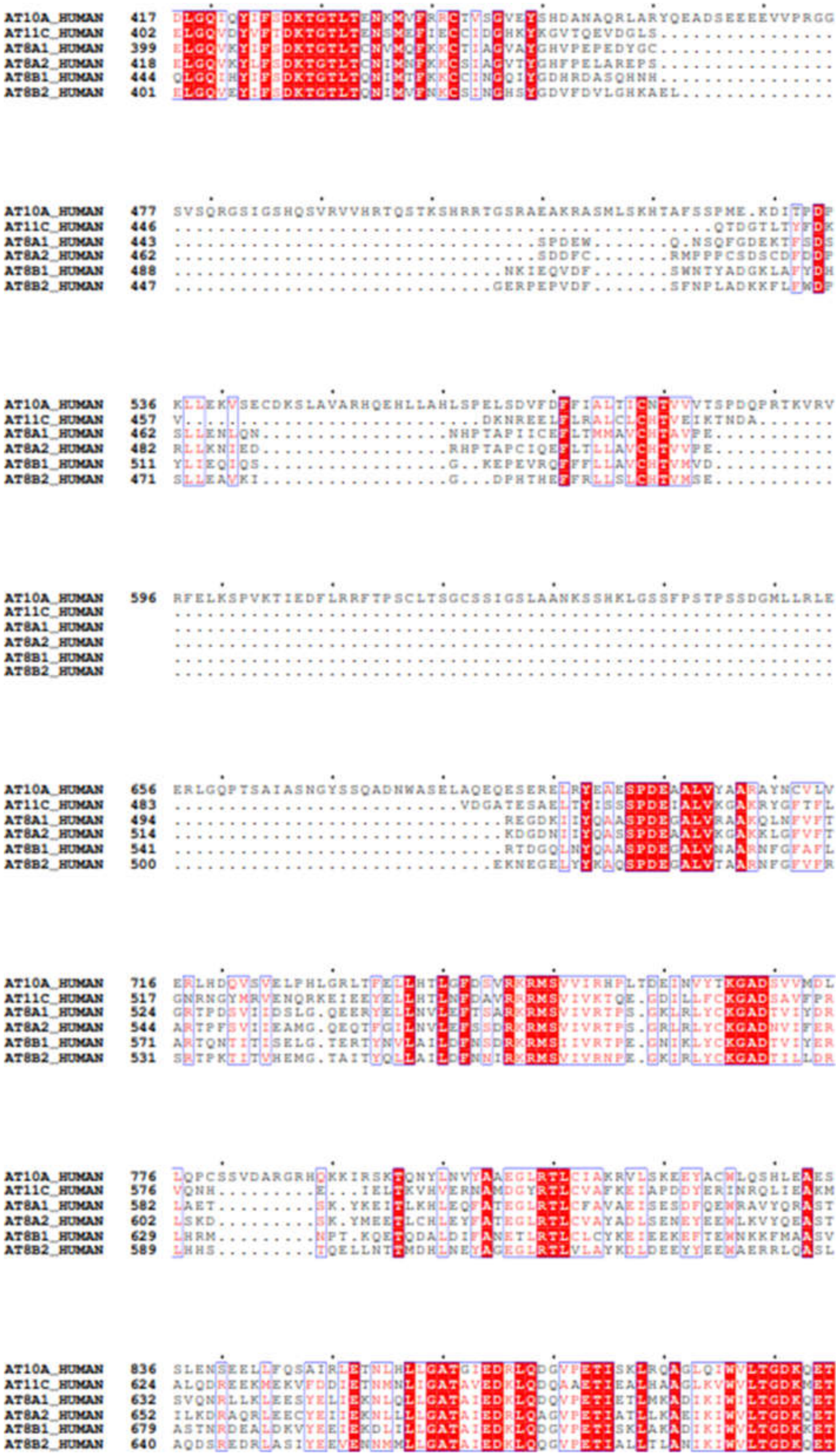
AT10A_HUMAN 417
AT11C_HUMAN 402
AT8A1_HUMAN 399
AT8A2_HUMAN 418
AT8B1_HUMAN 444
AT8B2_HUMAN 401
AT10A_HUMAN 477
AT11C_HUMAN 446
AT8A1_HUMAN 443
AT8A2_HUMAN 462
AT8B1_HUMAN 488
AT8B2_HUMAN 447
AT10A_HUMAN 536
AT11C_HUMAN 457
AT8A1_HUMAN 462
AT8A2_HUMAN 482
AT8B1_HUMAN 511
AT8B2_HUMAN 471
AT10A_HUMAN 596
AT11C_HUMAN
AT8A1_HUMAN
AT8A2_HUMAN
AT8B1_HUMAN
AT8B2_HUMAN
AT10A_HUMAN 656
AT11C_HUMAN 483
AT8A1_HUMAN 494
AT8A2_HUMAN 514
AT8B1_HUMAN 541
AT8B2_HUMAN 500
AT10A_HUMAN 716
AT11C_HUMAN 517
AT8A1_HUMAN 524
AT8A2_HUMAN 544
AT8B1_HUMAN 571
AT8B2_HUMAN 531
AT10A_HUMAN 776
AT11C_HUMAN 576
AT8A1_HUMAN 582
AT8A2_HUMAN 602
AT8B1_HUMAN 629
AT8B2_HUMAN 589
AT10A_HUMAN 836
AT11C_HUMAN 624
AT8A1_HUMAN 632
AT8A2_HUMAN 652
AT8B1_HUMAN 679
AT8B2_HUMAN 640

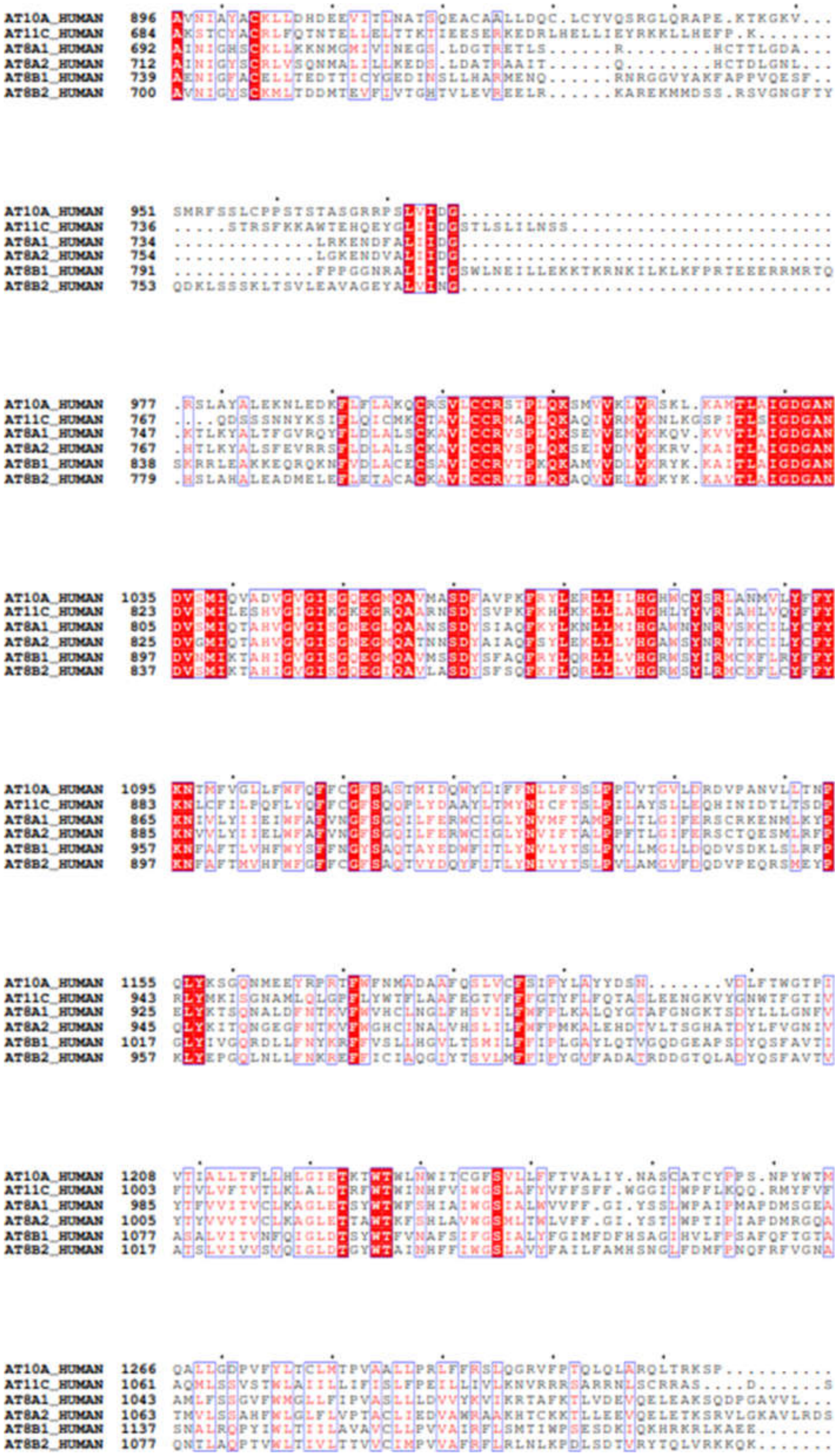

Figure S1. Sequence alignment of ATP8A1 and other P4-ATPase. UniProt accession numbers: ATP8A1 (UniProt: Q9Y2Q0), ATP8A2 (UniProt: Q9NTI2), ATP8B1 (UniProt: O4352), ATP8B2 (UniProt: P98198), ATP10A (UniProt: O60312), ATP11C (UniProt: Q8NB49). This alignment was performed with online server ENDscript (https://espript.ibcp.fr/ESPript/ENDscript/).

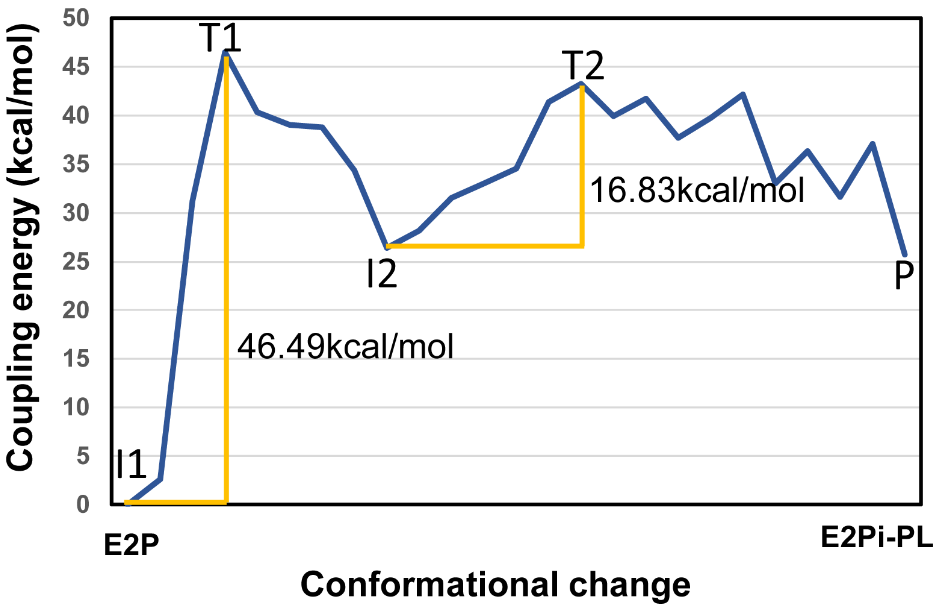

Figure S2. The coupling energy of the system during the conformational changes.

Table S1. The energy decomposition of I1, T1, I2, T2, P state (unit: kcal/mol).

	$E_{Form2MC}$ [1]	$E_{Scaled\ size}$ [2]	E_{Hydro} [3]	E_{VDW} [4]	$E_{-DG\ UF}$ [5]	E_{POLAR} [6]	E_{total} [7]	STD [8]
I1	−45.30	254.49	−946.28	−24.83	0.96	−66.02	−826.98	0.41
T1	−24.70	254.49	−923.85	−25.88	0.96	−63.30	−782.28	1.36
I2	−34.60	254.49	−943.07	−24.45	0.96	−66.52	−813.19	0.20
T2	−22.28	254.49	−943.83	−24.50	0.96	−67.30	−802.46	0.67
P	-19.53	254.49	-956.36	-24.00	0.96	-65.40	-809.84	0.40

[1] Electrostatic energy term obtained using whole residue charges (0 or ±1), which minimize electrostatic energy in the MCPT method. [2] Empirical term that takes into account the effect of protein size on folding free energy. [3] Scaled hydrophobic energy term. [4] Scaled van der Waals energy term. [5] Negative of a scaled charge–charge energy estimate of an unfolded protein. [6] Polar energy contribution term. [7] The sum of $E_{Form2MC}$, $E_{Scaled\ size}$, E_{Hydro}, E_{VDW}, $E_{-DG\ UF}$, and E_{POLAR}. [8] The standard deviation.
